# Gut microbiome communities demonstrate fine-scale spatial variation in a closed, island bird population

**DOI:** 10.1093/ismeco/ycaf138

**Published:** 2025-08-11

**Authors:** Sarah F Worsley, Chuen Zhang Lee, Maaike A Versteegh, Terry Burke, Jan Komdeur, Hannah L Dugdale, David S Richardson

**Affiliations:** School of Biological Sciences, University of East Anglia, Norwich Research Park, Norfolk NR4 7TJ, United Kingdom; School of Biological Sciences, University of East Anglia, Norwich Research Park, Norfolk NR4 7TJ, United Kingdom; Groningen Institute for Evolutionary Life Sciences (GELIFES), University of Groningen, P.O. Box 11103, 9700 CC, Groningen, The Netherlands; Ecology and Evolutionary Biology, School of Biosciences, University of Sheffield, Sheffield S10 2TN, United Kingdom; Groningen Institute for Evolutionary Life Sciences (GELIFES), University of Groningen, P.O. Box 11103, 9700 CC, Groningen, The Netherlands; Groningen Institute for Evolutionary Life Sciences (GELIFES), University of Groningen, P.O. Box 11103, 9700 CC, Groningen, The Netherlands; Faculty of Biological Sciences, School of Biology, University of Leeds, Leeds LS2 9JT, United Kingdom; School of Biological Sciences, University of East Anglia, Norwich Research Park, Norfolk NR4 7TJ, United Kingdom; Nature Seychelles, Roche Caiman, P.O. Box 1310, Mahé, Republic of Seychelles

**Keywords:** gut microbiome, biogeography, microbial ecology, environmental gradients, Acrocephalus sechellensis

## Abstract

Environmental variation is a key factor shaping microbial communities in wild animals. However, most studies have focussed on separate populations distributed over large spatial scales. How ecological factors shape inter-individual microbiome variation within a single landscape and host population remains poorly understood. Here, we use dense sampling of individuals in a natural, closed population of Seychelles warblers (*Acrocephalus sechellensis*) on Cousin Island (<0.7 km diameter, 0.34 km^2^ total area) to determine whether gut microbiome communities exhibit high-resolution spatial variation over fine scales (average territory area is 0.0023 km^2^). We identified a small but highly significant quadratic relationship between geographic distance and gut microbiome beta diversity across the island. Microbiome composition initially diverged with increasing geographic distance between territories. However, after ca. >300 m, microbiome composition became increasingly similar amongst individuals situated on different sides of the island. This relationship was robust to the effects of host relatedness, age, and sex. Further analysis showed that microbiome composition differed between individuals inhabiting coastal and inland territories. Warblers in coastal territories harboured greater abundances of marine bacteria and lower abundances of anaerobic taxa commonly linked to host metabolic health, suggesting that exposure to different environmental microbes and variation in host condition (which is lower in coastal territories) could drive spatial patterns of gut microbiome variation across the island. This work demonstrates that host–microbe interactions can be labile even at very fine spatial scales. Such variability may have implications for how species respond to anthropogenic disturbance in wild habitats.

## Introduction

The vertebrate gut microbiome plays an important role in host health by contributing to processes such as host digestion, behaviour, and immunity [1, 2]. However, in wild populations, gut microbiome composition can be extremely variable, even amongst individuals living in the same natural population [3–5]. In some cases, such variation has been associated with differences in host fitness components, including survival [6, 7], disease resistance [8, 9], and reproductive performance [10]. Thus, determining the drivers of inter-individual gut microbiome variation has important implications for understanding how host–microbe interactions shape the health and evolutionary trajectory of their hosts.

Various ecological factors have been proposed as drivers of gut microbiome variation in wild animal species. For example, variation in habitat type [11], anthropogenic disturbance [12], climatic variables (e.g. rainfall), and food availability [13] have all been associated with differences in microbiome composition. Such factors could have a direct impact on host–microbe interactions because variation in biotic and abiotic factors, coupled with microbial dispersal limitation, can lead to spatial heterogeneity in the pool of microbes able to colonize a host from the environment [14, 15]. Conversely, indirect effects could arise if the environment influences factors such as host condition, stress, and behaviour, all of which can alter the gut microbiome [16, 17].

The impact of environmental factors on the gut microbiome has primarily been demonstrated using host groups or populations that are distributed over large spatial scales (often separated by several to hundreds of kilometres) [e.g. 14, 18–20]. How ecological factors shape inter-individual gut microbiome variation at a much finer scale within a landscape (e.g. across territories that are metres apart), and within a single host population, is much less well understood. Indeed, although there is some evidence that geographic location can influence gut microbiome diversity within populations [11, 21, 22], most studies do not investigate these relationships in detail. Studying the role of environmental factors in shaping the microbiome at different spatial scales will not only shed light on the variability of host–microbe interactions but is especially urgent given the increasing influence of anthropogenic disturbance on wild habitats.

The microbiome of certain host taxa may be particularly sensitive to environmental variation. For example, birds and bats possess relatively short intestinal tracts as an adaptation to improve flight efficiency; this may increase the likelihood of these species acquiring microbial strains from their environment [23, 24]. However, few studies have investigated spatial variation in the microbiome of these taxa at fine spatial scales [but see 11, 21].

Here, we use an isolated population of Seychelles warblers (*Acrocephalus sechellensis*) on Cousin Island to understand how environmental factors influence the gut microbiome of individuals at fine spatial scales. Cousin Island measures <0.7 km in diameter (0.34 km^2^ total area) and is inhabited by ca. 320 adult Seychelles warblers, distributed across ca. 115 territories (average territory area 0.0023 km^2^) which are defended year-round [25, 26]. Territories differ in size, density, proximity to standing water, and quality (quantified in terms of insect abundance) across the island [27]. In particular, the prevailing wind direction, which differs between the two monsoonal seasons, can affect coastal territories as trees become defoliated by salt spray [27]. This can result in reduced insect abundances which have, in turn, been associated with reduced reproductive success in these territories [27].

Previous research has shown that individual Seychelles warbler gut microbiome composition varies according to season, average yearly territory quality, and host factors such as immunogenetic variation [5, 28, 29]. Gut microbiome variation has also been linked to differential survival [7, 29]. However, it is unknown whether spatial stratification of gut microbiome differences can be detected across the island. Here, we use dense sampling of individuals across the island to address this question. This dataset has previously been used to investigate longitudinal changes in the gut microbiome associated with host senescence [5]; however, we now combine this with detailed spatial information regarding each individuals breeding territory, as well as information on host genetic relatedness, to better understand how habitat features, individual location, and host factors combine to influence the gut microbiome. Very few studies have assessed spatial variation in the gut microbiome at such a fine scale [11, 21] and none with such a large and detailed dataset. Specifically, first we investigate whether gut microbiome diversity and composition differ according to the distance between territories on the island. We hypothesize that gut microbiome similarity will decrease at greater geographical distances due to spatial heterogeneity in habitat types and the abundances of environmentally derived microbial species, as has been observed at larger spatial scales [15, 19, 30]. Second, we investigate whether specific habitat features influence the gut microbiome. In particular, does habitat type (territories located on the exposed and sheltered coastline, or inland), distance to standing water, and/or territory connectivity impact gut microbiome characteristics? We expect microbiome differences between birds inhabiting coastal and inland territories due to differential exposure to marine microbes and potentially via indirect effects of inhabiting territories of differing quality (e.g. greater host stress and lower condition in coastal territories exposed to prevailing winds) [27, 31]. Distance to standing water may also impact the microbiome by influencing the availability of insect prey. Similarly, territory connectivity (the number of territories bordering each territory) may impact gut microbiome diversity via indirect territory quality effects if greater territory connectivity coincides with areas of higher quality habitat, but also due to the greater number of social interactions expected amongst birds living at greater density (e.g. via boundary disputes and extra-pair copulations) [32, 33].

## Materials and methods

### Study system

The Seychelles warbler is a small insectivorous passerine endemic to the Seychelles archipelago. Samples were collected from the closed population of warblers inhabiting Cousin Island (0.34 km^2^; 04°20′ S, 55°40′ E). This island is a nature reserve that is managed purely for conservation and research purposes with limited human disturbance. Virtually all warbler individuals are uniquely marked with a combination of a British Trust for Ornithology metal ring and three plastic colour rings, enabling identification and monitoring throughout their lives [34, 35]. Population monitoring is carried out biannually in the minor (January–March) and major (June–September) breeding seasons, respectively.

Territories consist of a dominant breeding pair which may also be accompanied by independent subordinates; subordinates are often (but not always) retained offspring from previous breeding seasons and may provide help in future reproductive attempts [25, 36]. Each sampling season, territories are extensively surveyed for the dominant breeding pair (identified through courtship and pair interactions) and subordinate individuals. Foraging occurs exclusively within the territory and defensive behaviours, including physical fights, are observed at territory boundaries [37, 38]. Each breeding season, individuals within each territory are followed for 15–30 min at least once a fortnight to observe territorial behaviours and accurately define territory boundaries. Territories are geographically located using a permanent 50 × 50-m grid of reference poles. Digital territory maps are subsequently generated using ArcGIS PRO software. The mean (±SE) territory size on Cousin Island is 0.0023 ± 0.0001 km^2^ [26].

### Habitat classification

**Figure 1:**
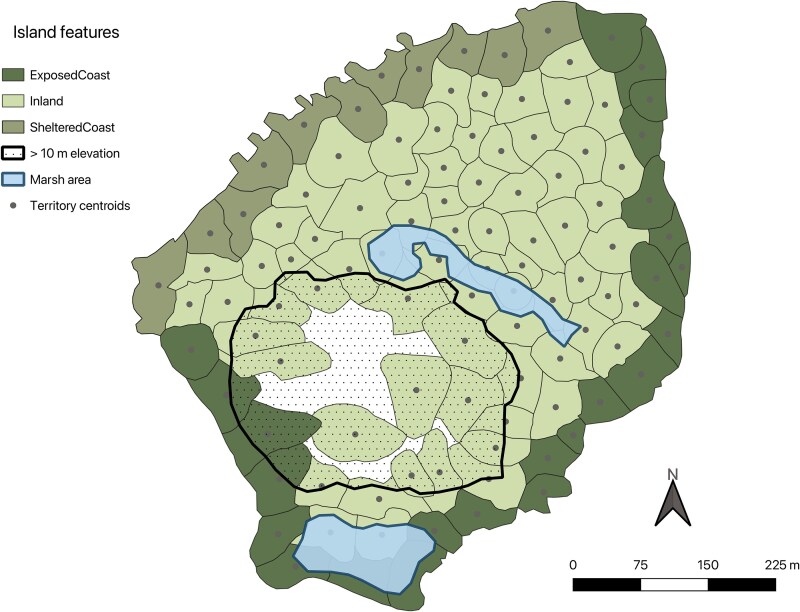
Seychelles warbler territories on Cousin Island. The 2021 major breeding season map is shown as an example. Territories have been coloured according to their habitat type. Prevailing winds come from the southeast in the major breeding season; thus, exposed coastal territories are those with a direct boundary on the southeast coast (note that the opposite is true in minor breeding seasons). Two marshy areas (where open water sometimes exists) have been marked in blue. An area >10 m elevation above sea level is also shown (dotted area). Territory centroids are marked with grey points.

Territories were classified into habitat categories using ArcGIS PRO software. The prevailing wind direction has a profound effect on coastal territories, whereby salt spray leads to the defoliation of vegetation and a subsequent reduction in insect abundances [27, 31, 39]. Prevailing winds come from the southeast (SE) in April–September or from the northwest (NW) in October–March. Thus, territories with a direct boundary on the SE coast were categorized as ‘exposed coast’ during the major breeding season, whilst the remaining coastal territories were categorized as ‘sheltered coast’ (Fig. 1). The opposite was the case for the minor season. All territories with no coastal border were classified as ‘inland’ (Fig. 1).

Two areas that are permanently marshy and can contain standing water are found on Cousin Island; one is a mangrove swamp dominated by grey/white mangrove (*Avicennia marina*) and the other is a freshwater marsh area (Fig. 1). Although the size of the freshwater marsh fluctuates greatly with rainfall, it is always marshy and normally contains some standing water year-round (Fig. 1). The distance of each territory to the freshwater marsh was calculated as the distance (metres) from the marsh edge to the centre of each territory. Distances ranged from 0 m (where the territory overlapped with the marsh) to 301 m.

The density of territories also differs across the island; local density is lower in the central elevated area (Fig. 1, >10 m elevation) which is rocky and has sparser vegetation compared to the central lowland plateau [27]. Territory density was calculated as the number of territories found within a 50-m radius of each territory (mean ± SE = 13.33 ± 0.13). Local territory connectivity was calculated as the number of territories sharing a physical border with each territory (mean ± SE = 4.85 ± 0.06). Connectivity was strongly correlated with territory density (Fig. S1). As such, only territory connectivity is included in downstream analyses.

### Sample collection

Faecal samples were collected across ten breeding seasons between 2017 and 2022. Each season, birds were caught in mist-nets and then placed into a disposable, flat-bottomed paper bag containing a sterilized weigh boat protected by a metal grate [28, 40]. This allows faecal matter to be collected from the tray whilst reducing contamination from the bird’s surface. Birds were removed from the bag after defecation or after 30 min. Faecal samples were collected using a sterile flocked swab and placed into a microcentrifuge tube containing 1 ml of absolute ethanol. Control swabs from fieldworker hands and collection bags were also taken at time of sampling. All samples were stored at 4°C for the remainder of the season (0–3.8 months) before being transferred to −80°C for long-term storage. A blood sample was also taken from each bird via brachial venipuncture and stored in absolute ethanol at 4°C. DNA was extracted from blood samples using the DNeasy Blood and Tissue kit (Qiagen, Crawley, UK); DNA was used for molecular sexing via a microbial ecology (PCR)-based method [35, 41] as well as genotyping at up to 30 polymorphic microsatellite loci [see 34, 35].

Fieldwork was carried out in accordance with local ethical regulations and agreements (University of East Anglia ethics approval ID ETH2223-0665). The Seychelles Department of Environment and the Seychelles Bureau of Standards approved the fieldwork (permit number A0157).

### Microbiome sequencing and bioinformatics

Genomic DNA was extracted from all faecal samples and collection controls using the DNeasy PowerSoil kit (Qiagen), according to a modified version of the manufacturer’s instructions [see 28]. Modifications included heating samples in the C1 buffer (65°C for 10 min) prior to bead beating and using 60 μl elution buffer. Extracted DNA was submitted for 16S rRNA gene amplicon sequencing at the NEOF Centre for Genomic Research (Liverpool, UK). Amplicon sequencing libraries were generated using the V4 primers 515F and 806R [see 28] and underwent 2 × 250-bp, paired-end sequencing on an Illumina MiSeq platform. Negative extraction blanks (ca. 1 per 50 samples) and a ZymoBIOMICS microbial mock community standard (D6300) were also sequenced to identify contaminants, check for batch effects, and assess sequencing success [as described in 5].

Sequencing reads were processed using QIIME2 2019.10 [42]. Briefly, forward and reverse reads were truncated at 240 bp and low-quality base calls were trimmed from the 5′ end using the DADA2 plugin [43]. Amplicon sequencing variants (ASVs) were then inferred for each sample, followed by dereplication and pair-end joining, as well as the removal of putative chimeras and singleton reads. ASVs were then taxonomically classified by training a naïve-Bayes classifier on the SILVA 138 reference database [44] for 16S rRNA gene sequences. ASVs classified as chloroplast or mitochondria were subsequently removed. A mid-point rooted phylogeny was constructed using MAFFT [45] and Fast Tree [46]. The final ASV, taxonomy, and tree files were exported from QIIME2 into R 4.2.2 for use in all subsequent analysis [47].

Files were further processed using *phyloseq* 1.42.0 [48]. ASVs were filtered to remove non-bacterial sequences and those unassigned at phylum level. Potential contaminants were also identified and removed from faecal samples using the prevalence method in *decontam* 1.18.0 [49]. This method identifies putative contaminants by detecting an increase in the prevalence of ASVs across negative controls compared to true samples. We ran the prevalence method in two steps by identifying ASVs with greater prevalence in blank extraction controls followed by collection controls, respectively. Following decontamination, samples with <8000 reads (27 samples) were removed based on rarefaction curves and samples reaching >95% completeness at this depth. Additionally, ASVs with <50 reads in total (across the entire dataset) were removed prior to downstream analysis as these may represent sequencing or clustering errors (filtered ASVs accounted for ~1% of all reads). A cut-off of 50 reads was chosen based on the presence of low abundance ASVs (<50 reads) in positive controls.

In total, 691 samples from 390 individuals were included in downstream analysis (sample sizes are summarized in Table S1). These were individuals classified as old fledglings (3–5 months of age), sub-adults (5 months to 1 year), or adults (≥1 year). We excluded samples from chicks (still in the nest) and fledglings (<3 months of age) due to small sample sizes; these individuals are also still dependent on their parents and harbour an immature gut microbiome [7]. A total of 21 029 ASVs were identified across the 691 samples (mean per sample 225.72 ± 6.11 SE).

### Statistical analysis

#### Alpha and beta diversity metrics

Samples were rarefied to 8000 reads (based on samples reaching >95% completeness at this depth) prior to calculating alpha diversity metrics. Observed ASV richness and Shannon diversity were calculated for each sample using *phyloseq* 1.42.0 [48]. Unrarefied reads were used to calculate gut microbiome beta diversity (i.e. compositional differences amongst samples). Unrarefied reads were filtered to remove rare taxa present in <5% of samples (78% of reads were retained). Microbiome datasets are extremely sparse, meaning that many ASVs are found in only a few samples. The power for these ASVs in statistical tests is extremely low and including them in analyses increases noise and the burden of correcting for multiple testing in differential abundance tests [50, 51]. This burden can be great enough as to prohibit detecting true associations [50, 51]. Thus, we took a more targeted approach (removing low prevalence taxa which are especially common in wild animal datasets) to improve the power of analyses. After prevalence filtering, ASV abundances were transformed using a centred log ratio (CLR) transform in *microbiome* 1.20.0 [52]. This approach takes unrarefied reads as an input, accounts for the compositional nature of microbiome datasets, and produces values that are scale invariant (i.e. not influenced by differences in library size across samples) [53]. Finally, a pairwise Aitchison distance matrix of CLR transformed ASV abundances (i.e. beta diversity) was constructed using *vegan* 2.6.8 [54].

#### Geographic distance and gut microbiome similarity

To establish whether warbler gut microbiome characteristics varied with geographic distances between territories, a series of distance matrices was constructed. To test whether alpha diversity similarity was higher amongst certain individuals (e.g. those sharing a territory/similar habitat types or individuals that were more closely related), a Euclidean distance matrix of sample alpha diversity (either Shannon diversity or observed ASV richness) was constructed using *vegan* 2.6.8 [54]. A matrix of geographic distances between territory centroids was calculated using the st_distance() function in *sf* 1.0.16 [55, 56]. Geographic distances ranged from 0 m (individuals in the same territory) to 698 m (average distance 283.43 ± 0.86 SE). The minimum distance between adjacent territory centroids (i.e. not including individuals in the same territory) was 25.81 m. Pairwise alpha diversity or Aitchison (beta diversity) gut microbiome distance matrices were unfolded into a dataframe of pairwise comparisons and used as the response variable in separate Multiple Regression on distance Matrices (MRM) models. Alpha diversity distances were right skewed and therefore square root transformed prior to analysis. In MRMs, tests of significance are performed using a randomized permutation procedure which controls for the non-independence of pairwise comparisons involving the same sample [57, 58]. MRMs were conducted using the MMRR function [implemented by 59] using 999 permutations.

Geographic distance was included as an independent variable in models. We also controlled for differences in sex (1 = same sex, 0 = different sex), age class (1 = same age class, 0 = different age class), and genetic relatedness between individuals. Pairwise genetic relatedness was calculated using *related* 1.0 [60] based on data from genotyping up to 30 microsatellite loci [34, 35] and the Queller and Goodnight’s estimation of relatedness [61]. We tested for nonlinear, quadratic relationships between geographic distance, as well as relatedness, and gut microbiome distances, but, in all models, quadratic terms (i.e. geographic distance^2^ and relatedness^2^) were removed sequentially if not significant (in order of least significant) to enable interpretation of the main effects. To simplify models and avoid the confounding effect of temporal environmental variation across sampling periods (which we know has a considerable effect on the gut microbiome [5]), we only included comparisons of samples taken from different individuals within the same sampling period (i.e. excluding between-sampling period and same individual comparisons). In total, 27 330 pairwise comparisons were included in the full model. To test whether observed relationships persisted over temporal scale and were not an artefact of combining several years of data (though only using pairwise comparisons of samples taken during the same sampling season), we also re-ran models using data from individual years (6 years) and sampling seasons (10 individual sampling seasons). Additionally, to further test whether habitat variation across the island was having an impact on the relationship between geographic and gut microbiome distances, we re-ran models using only inland–inland or coastal–coastal pairwise territory comparisons (i.e. excluding coastal or inland territories in turn; 11 522 and 3587 pairwise comparisons in each model, respectively). All variance inflation factors were <2, indicating no issues with collinearity.

#### Landscape features and gut microbiome differences

To further investigate the importance of habitat type and landscape features in driving variation in gut microbiome alpha diversity across individuals, generalized linear mixed models were constructed with either a Gaussian (for Shannon diversity) or negative binomial (for observed ASV richness) distribution using *lme4* 1.1.34 [62]. Habitat type (exposed coast, sheltered coast, or inland), distance to marsh, local territory connectivity, age, and sex (male/female) were included as predictors. We also controlled for the time of day at which samples were collected (minutes since sunrise) and the number of days samples were stored at 4°C in the field, both previously shown to impact the warbler gut microbiome [5]. Bird ID and sample year were included as random effects. We tested for quadratic relationships between distance to marsh, as well as local territory connectivity, and gut microbiome alpha diversity, but these quadratic terms were not significant and so were removed to enable interpretation of the main effects. We tested for residual spatial autocorrelation using the Moran’s *I* test embedded within *DHARMa* 0.4.6 [63]; however, this was not significant for any models, indicating that spatial variation had already been adequately explained by independent terms.

A marginal permutational analysis of variance (PERMANOVA) was used to test whether variation in gut microbiome beta diversity was associated with habitat type and landscape features. This was performed on pairwise Aitchison distances using the *adonis2()* function within *vegan* 2.6.8 [54]. The same predictors were used as for alpha diversity analysis. Bird ID was included as a blocking factor to control for repeated sampling. Differences in beta diversity were visualized using a principal components analysis (PCA) in *vegan* 2.6.8 [54]. The function betadisper was used to check for differences in group dispersion values [54].

To test whether the abundances of specific bacterial ASVs differed according to habitat type, an Analysis of Compositions of Microbiomes with Bias Correction (ANCOM-BC) was performed using *ancombc2* 2.1.4 [64]. Only landscape factors that were significantly associated with gut microbiome beta diversity were included as predictors in the model. However, host age, sex, time of day, storage time in the field, season, and sample year were controlled for in all analyses. Bird ID was included as a random effect. As part of ANCOM-BC, the Holm method was used to correct *P*-values for multiple testing. Example maps showing the average abundance per territory of the most differentially abundant ASVs were generated using *sf* 1.0.16 [55, 56] and *tmap* 3.3.4 [65] using the Cousin Island 2021 major breeding season territory map as a base layer.

## Results

### Geographic distance and gut microbiome similarity

Alpha diversity distances based on observed ASV richness were significantly lower amongst individuals within the same (versus different) age class (*P* = 0.009, Table S2; mean distance ± SE = 130.27 ± 0.99 and 1.33.46 ± 1.06 for individuals in the same or different age class, respectively) and, marginally, between individuals with higher relatedness scores (*P* = .045, Table S2). None of the other variables (geographic distance or sex differences) were associated with ASV richness distances between individuals (*P* > .05, Table S2). Gut microbiome distances based on sample Shannon diversity were not associated with any of the predictors in the model (*P* > .05, Table S2).

**Figure 2:**
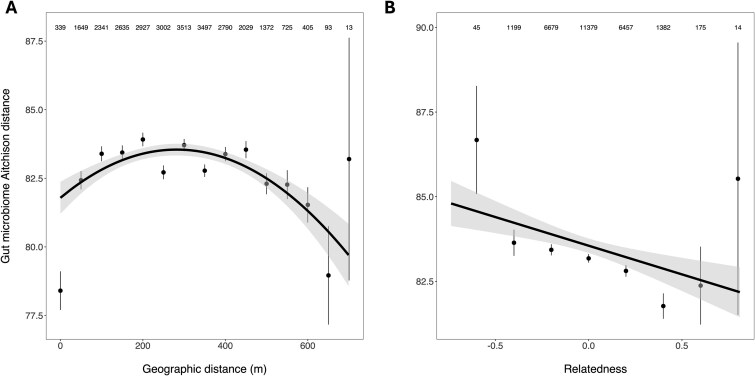
The relationship between (A) geographic distance (in metres) or (B) pairwise genetic relatedness and gut microbiome Aitchison distance (beta diversity) in Seychelles warblers. Points represent the mean (±SE) Aitchison distance per (A) 50 m or (B) 0.2 relatedness score and are calculated from the raw data. Numbers at the top of each panel represent the number of pairwise comparisons contributing to each mean. Total *N* = 27 330 pairwise comparisons between 691 samples (from 390 individuals) were included in models. Black lines are the model predicted slopes ±95% CI from a multiple regression on distance matrices (MRM) model (permuted *P*-values <.05).

There was a significant, quadratic, relationship between geographic distance and gut microbiome beta diversity on Cousin Island (*P* = .001, Fig. 2, Table 1). Specifically, gut microbiome composition was most similar (i.e. lowest Aitchison distances) amongst individuals sampled in the same territory (0 m geographic distance) but gradually diverged as the geographic distance between territories increased (Fig. 2). However, after ca. 300 m, gut microbiome composition became increasingly similar again as geographic distances increased (Fig. 2). This relationship largely persisted over temporal scale suggesting it was not an artefact of combining several years of data. Indeed, the quadratic relationship between geographic and gut microbiome distances was significant in five out of six years when analysed individually (Table S3, Fig. S2). A quadratic relationship was also evident in 4 out of 10 sampling seasons (Table S4, Fig. S3). Of the remaining sampling seasons, three showed a significant, positive linear relationship between geographic and gut microbiome distances and three had no significant relationship. However, inferences from individual sampling season subsets are severely limited by small sample sizes (sample sizes range = 29–115, Table S4) and, particularly, a lack of samples collected within the same territory and at the largest geographic distances within each season (Fig. S3).

**Table 1:** The results of a multiple regression on distance matrices (MRM) model investigating the relationship between geographic distance and gut microbiome beta diversity in Seychelles warblers

**Predictor**	**Estimate**	***t***	**Permuted *P***
Intercept	82.726	258.115	.721
**Geographic distance**	**0.012**	**5.733**	**.001**
**Geographic distance** ^**2**^	**<−0.001**	**−6.171**	**.001**
**Sex similarity**	**−0.367**	**−2.240**	**.024**
**Age similarity**	**−1.394**	**−8.491**	**.001**
**Relatedness**	**−1.693**	**−3.837**	**.001**

A total of 27 330 pairwise comparisons of 691 samples were included in the model. Reference categories were different sex (0) and different age class (0) for sex and age similarity variables, respectively. Tests of significance were performed using a randomized permutation procedure (999 permutations) to control for the non-independence of pairwise comparisons involving the same sample. Significant predictors are shown in bold.

The relationship between geographic and gut microbiome distances was robust, even after controlling for the relatedness of individuals in the model. This is despite there being a significant quadratic relationship between geographic distances and host genetic relatedness (*P* = .001, Fig. S4), whereby pairwise relatedness was highest amongst individuals in the same territory and those in territories that were furthest apart.

The greatest geographic distances on Cousin Island are between coastal territories on different sides of the island; as such, the quadratic relationship identified between gut microbiome and geographic distances (Table 1, Fig. 2) may partly reflect similarity in habitat type along the coast. To test whether differences in habitat type were having an impact on the relationship between geographic and gut microbiome distances, we re-ran the model using only coast–coast or inland–inland pairwise territory comparisons. In each case, this revealed a significant linear relationship, whereby gut microbiome distances increased with increasing geographic distance between territories (*P* = .004 for inland and .001 for coastal comparisons, respectively; Table S5, Fig. S5).

There was a significant, linear negative relationship between gut microbiome distances and the pairwise relatedness of individuals (*P* = .001, Fig. 2, Table 1). Similarly, gut microbiome compositional distances were significantly lower amongst individuals in the same age class and of the same sex (*P* = .001 and .024, respectively, Table 1).

### Landscape features and gut microbiome differences

Neither gut microbiome Shannon diversity nor observed ASV richness varied according to differences in territory habitat type (exposed coast, sheltered coast, or inland territories), distance to marsh, or with territory connectivity (*P* > .05, Table S6).

**Figure 3:**
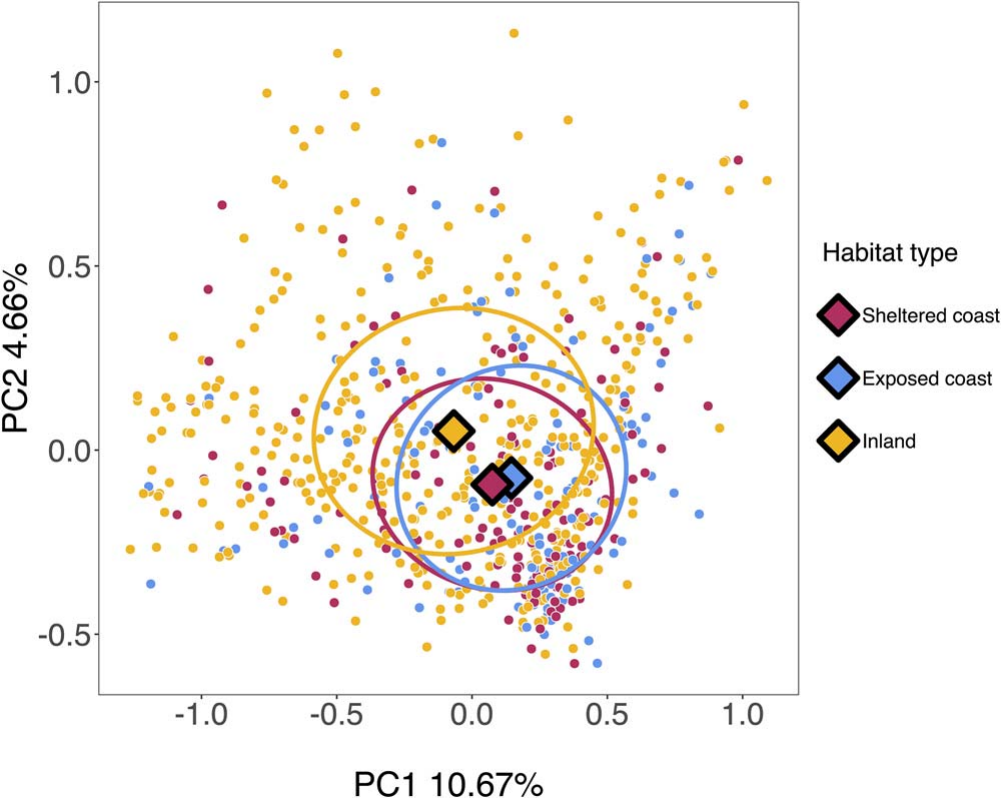
Variation in Seychelles warbler gut microbiome composition according to the habitat type of the individual’s territory on Cousin Island. The PCA ordination was carried out using Aitchison distances calculated on centred log ratio (CLR)–transformed amplicon sequencing variant (ASV) abundances. Each point represents a unique gut microbiome sample (*N* = 691 samples from 390 individuals). Large diamonds represent the group centroids and ellipses are the standard deviation of each centroid. Principal components 1 and 2 explained 10.67% and 4.66% of the variation in gut microbiome composition, respectively.

Gut microbiome composition varied significantly according to habitat type (*P* = .004, *R*^2^ = 0.008 in a PERMANOVA, Table 2). A PCA plot showed that the gut microbiome of individuals inhabiting coastal territories (both on the exposed and sheltered coast) tended to cluster away from those in territories situated inland (Fig. 3). A betadisper test also revealed that gut microbiome variation was greater amongst individuals inhabiting inland territories versus amongst warblers inhabiting coastal territories (*P* < .001 and *P* = .001 for inland versus exposed and sheltered coast territory comparisons, respectively, Fig. S6). Conversely, neither distance to marsh nor territory connectivity were significant predictors of gut microbiome composition (*P* > .05, Table 2). Bird age and sex were also not associated with gut microbiome composition (*P* > .05, Table 2). Year of sampling explained the largest proportion of variance in gut microbiome composition (*R*^2^ = 0.023, *P* < .01, Table 2, Fig. S7). The sampling season, time-of-day samples were collected, and the time stored at 4°C were also significantly associated with variation in gut microbiome composition (Table 2; *P* < .01, *R*^2^ = 0.004, 0.006, and 0.004, respectively).

**Table 2:** A PERMANOVA of the relationship between gut microbiome compositional differences and habitat features in Seychelles warblers

**Predictor**	***df***	***R*** ^**2**^	***F***	***P***
**Habitat type**	**2**	**0.008**	**2.980**	**.004**
Distance to marsh	1	0.003	1.881	.457
Territory connectivity	1	0.002	1.722	.525
Age	1	0.002	1.246	.979
Sex	1	0.002	1.543	.552
**Season**	**1**	**0.004**	**2.678**	**<.001**
**Sample year**	**5**	**0.023**	**3.300**	**<.001**
**Time of day**	**1**	**0.006**	**4.384**	**<.001**
**Storage time**	**1**	**0.004**	**2.719**	**.001**

The analysis was performed using Aitchison distances calculated using centred log ratio (CLR)–transformed amplicon sequencing variant (ASV) abundances. Significant predictors (*P* < .05) are shown in bold. *N* = 691 samples from 390 individuals. Bird ID was included as blocking factor to control for repeated measures.

### Differential abundance analysis

**Figure 4:**
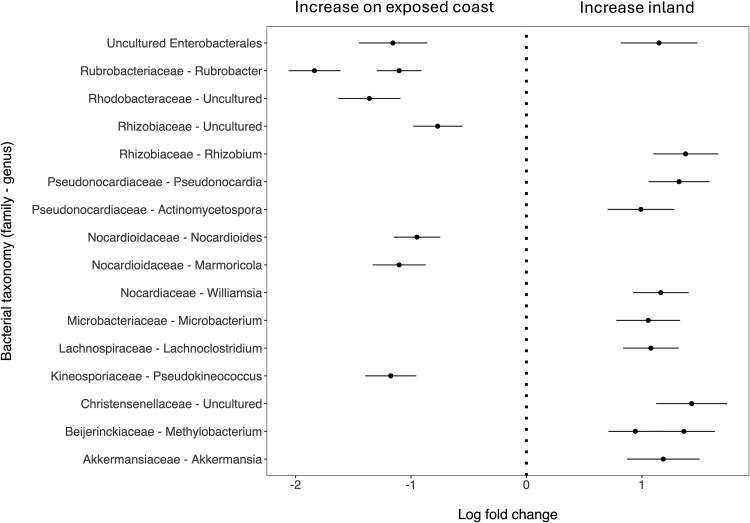
Differentially abundant amplicon sequencing variants (ASVs) in the gut microbiome of Seychelles warblers inhabiting exposed coastal versus inland territories on Cousin Island. *N* = 691 samples from 390 individuals were included in the analysis. Points represent the log fold change (effect size ± SE) of individual bacterial ASVs calculated using an ANCOM-BC model; only those with significant effect sizes (*P*_adj_ < .05) are shown. Multiple points per row indicate where more than one bacterial ASV per taxonomic group was significantly differentially abundant between habitat types. A positive log fold change indicates that an ASV is more abundant in individuals inhabiting inland territories (right), and a negative log fold change indicates a higher abundance in individuals in exposed coast territories (left). Results of differential abundance tests and full ASV taxonomies are presented in Table S7.

**Figure 5:**
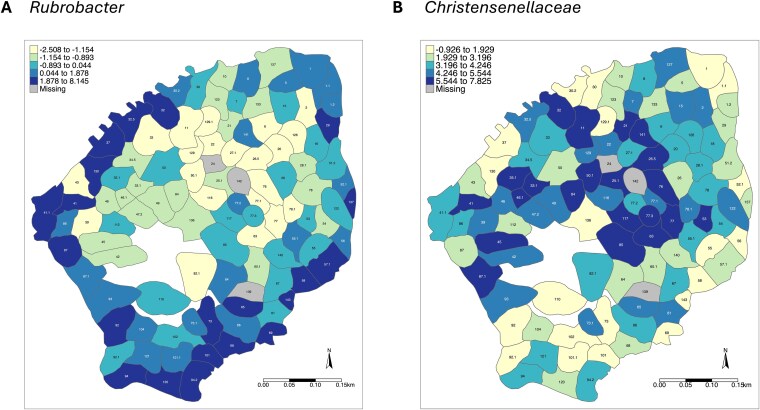
The average abundance of Seychelles warbler gut bacterial ASVs classified as (A) *Rubrobacter* or (B) *Christensenallaceae* across different territories on Cousin Island. Territories are coloured according to the mean abundance of each ASV per territory across all sampling periods (2017–2022). A centred log ratio transformation was applied to ASV abundances prior to averaging. The 2021 major season territory map is used as a base layer; numbers represent unique territory IDs. Territories with no gut microbiome samples are shown in grey (‘missing’). Plotted ASVs were identified as having significantly greater abundances (*P* < .05) in (A) exposed and sheltered coast territories and (B) inland territories, respectively (see Table S7 for full results of differential abundance tests using ANCOM-BC).

We next tested if specific ASVs differed in abundance between territory habitat types. In total, 19 ASVs were significantly differentially abundant between exposed coast and inland territories (*P*_adj_ < .05, Fig. 4, Table S7). Of these, eight ASVs were more abundant in exposed coast territories (Fig. 4, Table S7); three were in the phylum Proteobacteria (in the families *Rhodobacteraceae*, *Rhizobiaceae*, and one uncultured member of the order Enterobacterales) and five were in the phylum Actinobacteriota (two ASVs in the genus *Rubrobacter*, one in the genus *Pseudokineococcus*, one in the genus *Marmoricola*, and one in the genus *Nocardioides*). The remaining 11 ASVs were more abundant in inland (versus exposed coast) territories (Fig. 4, Table S7). Of these, one ASV was in the phylum *Verrucomicrobiota* (genus *Akkermansia*), four were in the phylum *Proteobacteria* (two in the genus *Methylobacterium*, one in the genus *Rhizobium*, and one uncultured member of the order *Enterobacterales*), two in the phylum *Firmicutes* (one in the genus *Lachnoclostridium* and one in the family *Christensenellaceae*), and four in the phylum *Actinobacteriota* (one in each of the genera *Actinomycetospora*, *Microbacterium*, *Williamsia*, and *Pseudonocardia*). The abundances of the most differentially abundant taxa—one *Rubrobacter* ASV that was more abundant in exposed and sheltered coastal territories and one *Christensenellaceae* ASV that was more abundant inland—are plotted on territory maps as an example of these relationships (Fig. 5).

Only one ASV was differentially abundant between sheltered and exposed coastal territories; an ASV in the genus *Microbacterium* was more abundant in sheltered coastal territories (*P*_adj_ < .05, Table S7). Five ASVs were differentially abundant between sheltered coastal and inland territories (*P*_adj_ < .05, Table S7); two ASVs classified as the genera *Marmoricola* and *Rubrobacter*, respectively, were more abundant in sheltered coast territories, whereas three ASVs in the genera *Actinomycetospora*, *Williamsia*, and *Lachnoclostridium* were more abundant in inland territories.

## Discussion

In this study, we evaluated fine-scale spatial heterogeneity in vertebrate host–gut microbe interactions across a landscape using a small island population of the Seychelles warbler. We identified a small but highly significant quadratic relationship between geographic distance and gut microbiome beta diversity which emerged over very small spatial scales (geographic distances of <0.7 km in total). This relationship was robust even when controlling for host relatedness, age, and sex differences, and was likely to be partly driven by variation in habitat types across the island. Indeed, habitat type had a strong effect on the warbler gut microbiome, whereby the gut microbiome of individuals sampled in coastal territories diverged from those inhabiting inland territories, both in terms of overall composition, variability, and the abundance of specific bacterial ASVs.

Biogeographic patterns in gut microbiome diversity have been noted previously in other systems. For example, in humans, gut microbiome similarity generally decreases with geographic distance within and between populations [66] and individuals living in shared spaces tend to harbour more similar microbial communities [67]. There is also some evidence of spatial microbiome variation in wild animals, with greater gut microbiome similarities occurring amongst sympatric versus allopatric species [68, 69], populations [19], and individuals [21, 30]. However, such studies have often been conducted over the scale of several or even thousands of kilometres. We now show that spatial gut microbiome patterns can also be detected at much finer spatial scales (where territories are tens of metres apart) within a single species.

Patterns of spatial gut microbiome variation may be partly driven by an increase in genetic relatedness amongst individuals living in familial groups and/or near one another. Indeed, studies in humans [70] and wild animals [71–73] have shown that related individuals tend to harbour more similar microbiome communities than pairs of unrelated individuals. These studies highlight the importance of host genetic differences in regulating microbiome composition. Pairwise genetic relatedness was a significant predictor of microbiome similarity between Seychelles warblers. There was also a significant quadratic relationship between geographic distance and host genetic relatedness. Pairwise relatedness was highest amongst individuals in the same and neighbouring territories; this pattern likely arises because subordinate individuals are often offspring that have been retained from previous breeding attempts [25, 36] and because extra-pair paternity frequently derives from males in adjacent territories [34, 74]. Host relatedness then declined with increasing geographic distance before increasing again at the greatest distances between territories. The driver of the latter increase is not clear but may be linked to natal dispersal. Almost all Seychelles warbler subordinates eventually disperse from their natal territory [75, 76] and, although dispersal is not directly linked to inbreeding avoidance [77], there is evidence that joining an unrelated group enables individuals to obtain reproductive benefits through co-breeding and eventual territory inheritance [76, 78]. The relationship between geographic distance and host genetic similarity may partly drive an increase in gut microbiome similarity for individuals living in the same breeding group and at the greatest geographic distances. However, the negative quadratic relationship between geographic and gut microbiome distances remained even after controlling for relatedness in models. This suggests that additional factors may also be important in driving spatial patterns of gut microbiome variation in this species.

Recent research has also shown that microbial taxa can be shared amongst individuals via social interactions [32, 33, 79]. Microbial sharing can occur via direct interaction or through host microbial shedding to a shared environment [32]. Such processes could partly explain the increase in gut microbiome similarity amongst Seychelles warblers inhabiting the same territory. However, it is difficult to disentangle the relative importance of greater social transmission, versus increased relatedness and shared environmental conditions, when species are highly territorial. In the warbler, local territory connectivity may represent a proxy for the number of opportunities to interact with birds from neighbouring territories. For example, these interactions may occur via boundary disputes [37, 38] and extra-pair copulations with males from adjacent territories [34, 74]. However, local territory connectivity was not associated with gut microbiome composition in the Seychelles warbler. Furthermore, it is unlikely that social transmission dynamics are driving the gradual convergence of gut microbiome communities at the greatest geographic distances. Thus, although social interactions may play a role in structuring spatial gut microbiome variation, particularly amongst adjacent territories, other factors are also likely to be important.

On Cousin Island, the greatest geographic distances between Seychelles warbler territories arise between coastal locations (i.e. territories on different sides of the island). Thus, the gradual increase in microbiome similarity at distances ca. >300 m suggests that habitat similarity may be a key driver of compositional microbiome similarity. Indeed, habitat type was significantly associated with gut microbiome composition, whereby individuals in coastal territories harboured significantly different microbial communities compared to those inland. Comparisons between individuals in exposed coast versus inland territories yielded the greatest number of differentially abundant ASVs. However, contrasts between sheltered coast and inland territories identified some of the same significant taxa suggesting a general effect of coastal conditions on the gut microbiome that was made more extreme by the prevailing wind direction. Microbiome variation was also greater amongst individuals inhabiting inland, versus coastal, territories suggesting that coastal conditions may induce homogenizing, deterministic changes to the microbiome.

Fine-scale environmental differences between coastal and inland territories are likely to be a key factor driving spatial patterns of gut microbiome variation across Cousin Island. Indeed, environmental variation has been shown to be an important factor shaping the gut microbiome in captive cross-foster experiments [80, 81] and amongst other wild animal populations and individuals [11, 18, 82]. Whilst environmental differences could influence the microbiome indirectly, e.g. via effects on host stress and condition [17, 83], abiotic/biotic variation can also determine the type of microbes that can survive and be acquired by hosts horizontally from the external environment [15, 84]. Such acquisition may occur through direct interaction with the local environment, or indirectly, e.g. via variation in microbes derived from the host’s diet.

Diet is a key factor shaping the gut microbiome since it selects for microbial species with the metabolic capabilities to degrade and detoxify specific dietary components. As such, host species occupying distinct feeding guilds (e.g. herbivores versus omnivores and carnivores) have been shown to harbour distinct gut microbiomes [23, 85, 86]. Seychelles warblers are almost exclusively insectivorous; however, it is possible that the availability and type of insect prey varies according to habitat type across the island. This could influence the gut microbiome, either by selecting for specific microbes or because different prey items harbour distinct symbionts that, in turn, enter the gut ecosystem as transients. Passerines may be particularly susceptible to the latter since their short intestinal tracts (an adaptation to flight) can allow transient microbial species to persist during gut transit [23, 24]. Faecal DNA metabarcoding using specific primer sets (e.g. those targeting the mitochondrial cytochrome C oxidase subunit 1 locus) can provide information on the diversity and composition of arthropod diets [87–89]. In future work, such techniques could be used to shed further light on diet–microbiome relationships and the influence of habitat type on the gut microbiome of the Seychelles warbler.

Individuals inhabiting coastal territories generally harboured greater abundances of aerobic, marine-associated or extremophile bacterial taxa. For instance, members of the genus *Rubrobacter* are frequently isolated from marine environments and are tolerant of high levels of radiation, temperature, and salinity [90, 91]. Similarly, members of the *Rhodobacteraceae* and *Pseudokineococcus* have also been isolated from marine and hyper-saline environments [92, 93]. By contrast, individuals inhabiting inland territories tended to harbour greater abundances of bacterial taxa commonly found in soil and terrestrial habitats such as *Methylobacterium*, *Pseudonocardia*, and members of the *Rhizobiaceae* [94–96]. This suggests that differential exposure to, and acquisition of, environmental microbes may be driving some of the gut microbiome differences observed between inland and coastal birds. However, several anaerobic bacterial taxa that are commonly found in the gut microbiome of other vertebrate species were also more abundant in warblers inhabiting inland territories. This included ASVs in the genera *Lachnoclostridium*, *Enterobacteriaceae*, *Akkermansia*, and *Christensenellaceae*. Commensal members of the *Lachnoclostridium* (recently reclassified as *Clostridium*) and the *Enterobacteriaceae* play an important role in producing short-chain fatty acids such as butyrate and lactic acid [97–99]. Similarly, *Christensenallaceae* is one of the most heritable members of the human gut microbiome and the abundance of this family, as well as the genus *Akkermansia*, is positively associated with various aspects of metabolic health in mammals [100, 101]. Whilst the function of these taxa has not been assessed in passerines, and it is extremely difficult to disentangle cause from effect in wild systems, it is possible that differences in their abundance are linked to variation in dietary quality and the reduced condition of Seychelles warblers (evidenced by reduced reproductive success) living in exposed coast versus inland territories [27]. Further investigation of diet–microbiome relationships as well as functional characterization of gut microbes in avian species (e.g. using metagenomics and experimental disruption of the gut microbiome [102]) would be needed to understand whether this is the case.

Aside from habitat type, several other factors were found to be associated with variation in the Seychelles warbler gut microbiome, including sampling year and season. Indeed, differences in sampling year explained the greatest amount of variation in gut microbiome composition. This is consistent with previous work on the Seychelles warbler and is likely to be associated with variation in environmental conditions over time [103]. Seasonal gut microbiome variation could additionally be linked to host physiological changes associated with reproduction, whereby the majority of warblers reproduce in the major breeding season (June–September) [104, 105]. Despite yearly and seasonal microbiome variation, spatial patterns were found to be broadly consistent even when data were subsetted to individual sampling periods suggesting that spatial trends largely persist over temporal scale.

Although the relationship between several factors (e.g. geographic distance, relatedness, habitat type, and sampling year) and gut microbiome similarity were highly significant, it is important to note that each of these variables only explained a very low proportion of the overall variance in the Seychelles warbler gut microbiome composition. This suggests that drivers of gut microbiome variation remain poorly characterized in this system. However, such small effects are not unusual in wild animal microbiome studies [e.g. 3, 33]. For example, a large study on wild baboons identified very small (but highly significant) differences in gut microbiome Aitchison similarity amongst individuals living within the same or different social groups [3]. Longitudinal sampling of individuals in this system demonstrated that gut microbiome dynamics can be highly personalized over time [3]. Such individuality can create noise in microbiome datasets leading to only modest effects of unifying factors, such as shared environment and diet, on gut microbiome composition [3]. These processes could be particularly pronounced in passerines as an increased susceptibility to acquiring transient environmental microbes may lead to greater levels of intra- and inter-individual gut microbiome heterogeneity [23, 24].

In conclusion, our study demonstrates that gut microbiome communities can vary at extremely fine spatial scales within a landscape and that at least some of this variation is likely to be driven by differences in local environmental conditions. Further work is needed to understand the mechanisms by which the environment shapes the microbiome and the impact of spatial gut microbiome variation on host fitness, but this work suggests that host–microbe interactions can be extremely labile even amongst individuals of the same species living in close proximity. Given the importance of the gut microbiome to host health, such variability may have implications for the resilience of species to anthropogenic disturbance in wild habitats. This may be especially important in restricted, small island populations that have no emi- or immigration.

## Supplementary Material

FinalSupplementary20250802_ycaf138

## Data Availability

All sequencing reads have been uploaded to the European Nucleotide Archive (ENA) under the following accession numbers: PRJEB45408 (samples taken in 2017 and 2018), PRJEB47095 (samples taken in 2019 and 2020), and PRJEB67634 (samples taken in 2021 and 2022). The scripts and metadata to reproduce all analyses and figures can be accessed via the GitHub repository (https://github.com/Seychelle-Warbler-Project).
