# Supplementary material for: Gut microbiome communities demonstrate fine-scale spatial variation in a closed, island bird population
Source: ISME Commun. 2025 Aug 11;5(1):ycaf138. doi: 10.1093/ismeco/ycaf138 (PMC12400925; doi:10.1093/ismeco/ycaf138)

Supplementary Material

Table S1. The number of Seychelles warbler faecal samples per variable used in statistical models.

Variable	Category	Sample size
**Habitat type**	Inland	133
	Exposed coast	429
	Sheltered Coast	129
**Sample year**	2017	65
	2018	144
	2019	109
	2020	71
	2021	151
	2022	151
**Season**	Major	428
	Minor	263
**Ageclass**	Old fledgling	52
	Sub-adult	172
	Adult	467
**Sex**	Male	380
	Female	311

Table S2. The results of a Multiple Regression on distance Matrices (MRM) analysis investigating the relationship between geographic and gut microbiome alpha diversity distances in Seychelles warblers. Two metrics of alpha diversity – A) Shannon and B) observed ASV richness – were used to calculate Euclidean distance matrices that were included as the response variable in separate models. A total of 27,330 pairwise comparisons of 691 samples were included in each model. Reference categories were different sex (0) and different age class (0) for sex and age similarity variables, respectively. Tests of significance were performed using a randomized permutation procedure (999 permutations) to control for the non-independence of pairwise comparisons involving the same sample. Significant predictors are shown in bold and underlined.

**Predictor**	**Estimate**		***t***		**Permuted *P***	
	**A) Shannon**	**B) Richness**	**A) Shannon**	**B) Richness**	**A) Shannon**	**B) Richness**
Intercept	1.070	10.472	145.738	131.399	0.590	0.095
Geographic distance	<-0.001	<-0.001	-0.659	-0.165	0.568	0.858
Sex similarity	-0.002	-0.008	0.304	-0.136	0.765	0.900
Age similarity	0.008	<u>**-0.161**</u>	1.457	<u>**-2.701**</u>	0.151	<u>**0.010**</u>
Relatedness	<0.001	**-0.325**	0.128	**-2.034**	0.910	**0.045**

Table S3. The results of analyses investigating the relationship between geographic distance and Seychelles warbler gut microbiome beta diversity in individual years. Analyses were conducted using a Multiple Regression on distance Matrices (MRM) framework on yearly subsets of the dataset (2017-2022). N= 65, 144, 109, 71, 151 and 151 samples were included in the analysis for each of 2017, 2018, 2019, 2020, 2021, and 2022, respectively. The top number in each cell represents the model coefficient and the bottom value is the permuted *P*-value for each term. Tests of significance were performed using a randomised permutation procedure (999 permutations) to control for the non-independence of pairwise comparisons involving the same sample. Significant predictors are shown in bold. The quadratic of geographic distance was removed from models when not significant to enable interpretation of the main effects (represented by a dash for 2017). Reference categories were different sex (0) and different age class (0) for sex and age similarity variables, respectively.

	Sample Year					
**Predictor**	**2017**	**2018**	**2019**	**2020**	**2021**	**2022**
Intercept	77.968<br>0.664	83.080<br>0.990	85.494<br>0.345	91.323<br>0.991	84.259<br>0.010	74.749<br>1.000
Geographic distance	**0.005**<br>**0.015**	**0.001**<br>**0.013**	**0.022**<br>**0.001**	**0.015**<br>**0.010**	0.007<br>0.100	**0.014**<br>**0.002**
Geographic distance^2	-	**< -0.001**<br>**0.030**	**< -0.001**<br>**0.001**	**< -0.001**<br>**0.031**	**< -0.001**<br>**0.017**	**< -0.001**<br>**0.011**
Sex similarity	0.109<br>0.848	-0.318<br>0.276	-0.355<br>0.470	-0.368<br>0.393	**-1.248**<br>**0.002**	**0.853**<br>**0.011**
Age similarity	**-1.779**<br>**0.004**	**0.716**<br>**0.023**	**-2.170**<br>**0.001**	-0.350<br>0.440	**-1.521**<br>**0.001**	**1.260**<br>**0.001**
Relatedness	1.486<br>0.335	**-4.811**<br>**0.001**	**-4.072**<br>**0.002**	0.317<br>0.785	0.030<br>0.966	-0.300<br>0.738
**Sample size**	65	144	109	71	151	151
**Pairwise comparisons**	2076	6945	3066	2483	6729	6031

Table S4. The results of analyses investigating the relationship between geographic distance and Seychelles warbler gut microbiome beta diversity in individual sampling seasons. Analyses were conducted using a Multiple Regression on distance Matrices (MRM) framework individually for each sampling season. The top number in each cell represents the model coefficient and the bottom value is the permuted *P*-value for each term. Tests of significance were performed using a randomised permutation procedure (999 permutations) to control for the non-independence of pairwise comparisons involving the same sample. Significant predictors are shown in bold. The quadratic of geographic distance was removed from models when not significant to enable interpretation of the main effects (represented by a dash). Reference categories were different sex (0) and different age class (0) for sex and age similarity variables, respectively. The number of samples and pairwise comparisons included for each sampling season are given at the bottom of the table.

	Sampling Season ID									
**Predictor**	**164**	**166**	**167**	**171**	**173**	**174**	**175**	**176**	**177**	**178**
Intercept	77.968<br>0.691	78.704<br>0.656	84.019<br>0.969	89.347<br>0.259	72.846<br>0.998	91.323<br>0.991	73.403<br>1.000	**86.154**<br>**0.001**	74.599<br>0.686	81.679<br>0.893
Geographic distance	**0.005**<br>**0.014**	0.032<br>0.052	**0.002**<br>**0.026**	-0.001<br>0.549	0.005<br>0.052	**0.015**<br>**0.010**	**-0.044**<br>**0.001**	-0.002<br>0.078	**0.014**<br>**0.003**	**0.005**<br>**0.027**
Geographic distance^2	-	**<0.001**<br>**0.026**	-	-	-	**<0.001**<br>**0.029**	**<0.001**<br>**0.001**	-	**<0.001**<br>**0.001**	-
Sex similarity	0.109<br>0.864	-0.680<br>0.599	-0.248<br>0.396	-0.460<br>0.394	-1.162<br>0.214	-0.368<br>0.415	-0.781<br>0.370	**-1.321**<br>**0.001**	**1.325**<br>**0.001**	0.198<br>0.751

Age similarity	**-1.779** **0.003**	-1.549 0.213	**0.901** **0.004**	0.328 0.548	**5.537** **0.001**	-0.350 0.385	1.473 0.097	**-2.034** **0.001**	**-2.143** **0.001**	-
Relatedness	1.486 0.335	-5.191 0.078	**-4.593** **0.001**	0.437 0.746	**-8.783** **0.001**	0.317 0.796	-3.837 0.109	0.650 0.511	**-3.642** **0.001**	**8.329** **0.001**
**Sample size**	65	29	115	67	42	71	42	109	96	55
**Pairwise comparisons**	2076	406	6539	2209	857	2483	861	5868	4550	1481

Table S5. The results of a Multiple Regression on distance Matrices (MRM) model investigating the relationship between geographic distance and gut microbiome beta diversity across A) inland territories and B) coastal territories of the Seychelles warblers. A total of 11,522 inland and 3587 coastal pairwise comparisons were included in each model, respectively. Reference categories were different sex (0) and different age class (0) for sex and age similarity variables, respectively. Tests of significance were performed using a randomized permutation procedure (999 permutations) to control for the non-independence of pairwise comparisons involving the same sample. Significant predictors are shown in bold and underlined.

Predictor	Estimate	*t*	Permuted *P*
**A) Inland pairwise comparisons**			
Intercept	85.004	275.814	0.150
**Geographic distance**	**0.003**	**3.154**	**0.004**
Sex similarity	-0.268	-1.145	0.243
**Age similarity**	**-1.398**	**-5.980**	**0.001**
**Relatedness**	**-1.423**	**-2.298**	**0.019**
**B) Coastal pairwise comparisons**			
Intercept	75.854	114.868	1.000
**Geographic distance**	**0.008**	**6.075**	**0.001**
Sex similarity	-0.788	-1.606	0.110
Age similarity	0.229	0.458	0.644
Relatedness	-2.180	-1.687	0.085

Table S6. Variation in gut microbiome A) Shannon diversity and B) observed ASV richness according to territory habitat type and landscape features in Seychelles warblers. Estimates are derived from (generalised) linear mixed models with a gaussian or negative binomial distribution, respectively. A total of 691 samples from 380 individuals were included in each analysis. Significant predictors ($P < 0.05$) are shown in bold and underlined. The reference categories for categorical variables are as follows: exposed coast (habitat type), female (sex), major (season).

**A) Shannon diversity**				
**Predictor**	**Estimate**	**SE**	***t***	***P***
Intercept	3.200	0.141	22.670	<0.001
Habitat type				
Inland	0.032	0.143	0.223	0.824
ShelteredCoast	-0.019	0.157	-0.119	0.906
Distance to marsh	0.010	0.104	0.098	0.922
Territory connectivity	0.094	0.113	0.824	0.410
Age	-0.123	0.096	-1.277	0.202
Sex	-0.158	0.095	-1.675	0.037
Season	-0.069	0.120	-0.577	0.566
Time of day	-0.079	0.096	-0.827	0.408
**Storage time at 4°C**	**<u>-0.296</u>**	**<u>0.101</u>**	**<u>-2.924</u>**	**<u>0.004</u>**
**Random effects**	**691 Observations**		**Variance**	
Bird ID	390 individuals		0.060	
Sample Year	6 years		0.023	
**B) Observed ASV richness**				
**Predictor**	**Estimate**	**SE**	***z***	***P***
Intercept	5.234	0.115	45.521	<0.001
Habitat type				
Inland	0.148	0.081	1.833	0.067
Sheltered coast	0.091	0.089	1.023	0.306
Distance to marsh	-0.020	0.057	-0.354	0.723
Territory connectivity	0.067	0.065	1.038	0.299
Age	-0.041	0.055	-0.746	0.456
Sex	-0.100	0.053	-1.867	0.062
Season	-0.041	0.080	-0.510	0.610
Time of day	<-0.001	0.054	-0.005	0.965
**Storage time at 4°C**	**<u>-0.119</u>**	**<u>0.060</u>**	**<u>-1.977</u>**	**<u>0.048</u>**
**Random effects**	**691 Observations**		**Variance**	
Bird ID	390 individuals		0.011	
Sample Year	6 years		0.046	

Table S7. The results of an ANCOM-BC analysis investigating differences in gut microbiome amplicon sequencing variant (ASV) abundance according to territory habitat types in the Seychelles warbler. Amplicon sequencing variants (ASVs) that were significantly, differentially abundant ($P_{adj} < 0.05$) between two habitat categories are shown. Effect sizes (log fold change- "LFC") are shown with standard errors (SE). All *P*-values were adjusted with the Holm correction for multiple testing. A positive log fold change indicates that an ASV is more abundant in individuals inhabiting A) inland (versus exposed coast) territories B) Sheltered (versus exposed) coast territories and C) Sheltered (versus inland) territories. ASV taxonomic classifications are shown to bacterial genus level (or the highest resolution classification if unclassified at genus level).

ASV ID	LFC	SE	P_{adj}	Phylum	Family	Genus
**A) Exposed coast versus inland territories**						
f4007e7d3694c53b27b5828b2a971b63	-1.836	0.225	<0.001	*Actinobacteriota*	*Rubrobacteriaceae*	*Rubrobacter*
2e5c86b8abc011cf866470d59d4e783f	-1.361	0.269	<0.001	*Proteobacteria*	*Rhodobacteraceae*	
144d2b8f94ec382e34460e8217d6a750	-1.176	0.221	<0.001	*Actinobacteriota*	*Kineosporiaceae*	*Pseudokineococcus*
aee9f354c80ca7baa872c3da2fe462c2	-1.157	0.296	0.006	*Proteobacteria*		
ac01fd659628399d0265775786cad38e	-1.103	0.194	<0.001	*Actinobacteriota*	*Rubrobacteriaceae*	*Rubrobacter*
fac1e2799f556a919b52ebfb8ab31e5a	-1.102	0.231	<0.001	*Actinobacteriota*	*Nocardioidaceae*	*Marmoricola*

5df7f6da7fe2d484098b5119cb19028b	-0.949	0.202	<0.001	*Actinobacteriota*	*Nocardioidaceae*	*Nocardioides*
664765dce1a593784085345685a67650	-0.769	0.214	0.020	*Proteobacteria*	*Rhizobiaceae*	
e0f50c5adf537a0a3a63e61720b38ed5	0.943	0.233	0.003	*Proteobacteria*	*Beijerinckiaceae*	*Methylobacterium*
1c7a5248b18573f28b2901a63298dbc7	0.991	0.289	0.035	*Actinobacteriota*	*Pseudonocardiaceae*	*Actinomycetospora*
e4c616e0e34cf5f3837203933c18e498	1.055	0.276	0.008	*Actinobacteriota*	*Microbacteriaceae*	*Microbacterium*
09a261cd2ba6d5db1a38bfe0ef012286	1.078	0.240	<0.001	*Firmicutes*	*Lachnospiraceae*	*Lachnoclostridium*
3b7cb4615c07aeaa9415acee19c6db7c	1.149	0.332	0.032	*Proteobacteria*		
749906e6079c81c5b2979a147e503684	1.164	0.241	<0.001	*Actinobacteriota*	*Nocardiaceae*	*Williamsia*
574d387c22a18447c5c5375cf1f1b98d	1.185	0.314	0.010	*Verrucomicrobiota*	*Akkermansiaceae*	*Akkermansia*
ff933fa75ad5cc076102ec14b504da6c	1.323	0.265	<0.001	*Actinobacteriota*	*Pseudonocardiaceae*	*Pseudonocardia*
04ecfad5772d2e09a84a0f5ef460536c	1.363	0.270	<0.001	*Proteobacteria*	*Beijerinckiaceae*	*Methylobacterium*
d4ed8e95671bb076cf81536b9db1e1be	1.379	0.281	<0.001	*Proteobacteria*	*Rhizobiaceae*	*Rhizobium*

e938e5e953b3a9b73e97 a9197cd3a890	1.432	0.308	<0.001	*Firmicutes*	*Christensenellaceae*	
**B) Sheltered versus exposed coast territories**						
e4c616e0e34cf5f38 37203933c18e498	1.315	0.345	0.008	*Actinobacteriota*	*Microbacteriaceae*	*Microbacterium*
**C) Sheltered coast versus inland territories**						
1c7a5248b18573f28 b2901a63298dbc7	-1.423	0.337	0.002	*Actinobacteriota*	*Pseudonocardiaceae*	*Actinomycetospora*
749906e6079c81c5 b2979a147e503684	-1.326	0.296	<0.001	*Actinobacteriota*	*Nocardiaceae*	*Williamsia*
09a261cd2ba6d5db 1a38bfe0ef012286	-1.115	0.296	0.001	*Firmicutes*	*Lachnospiraceae*	*Lachnoclostridium*
fac1e2799f556a919b52 ebfb8ab31e5a	1.021	0.287	0.023	*Actinobacteriota*	*Nocardioidaceae*	*Marmoricola*
f4007e7d3694c53b 27b5828b2a971b63	1.243	0.282	0.001	*Actinobacteriota*	*Rubrobacteriaceae*	*Rubrobacter*

Figure S1. The correlation between territory connectivity and territory density for Seychelles warblers inhabiting Cousin Island. Territory connectivity is the number of territories sharing a physical border with each individual territory. Territory density is the number of territories found within a 50 m radius of each territory.

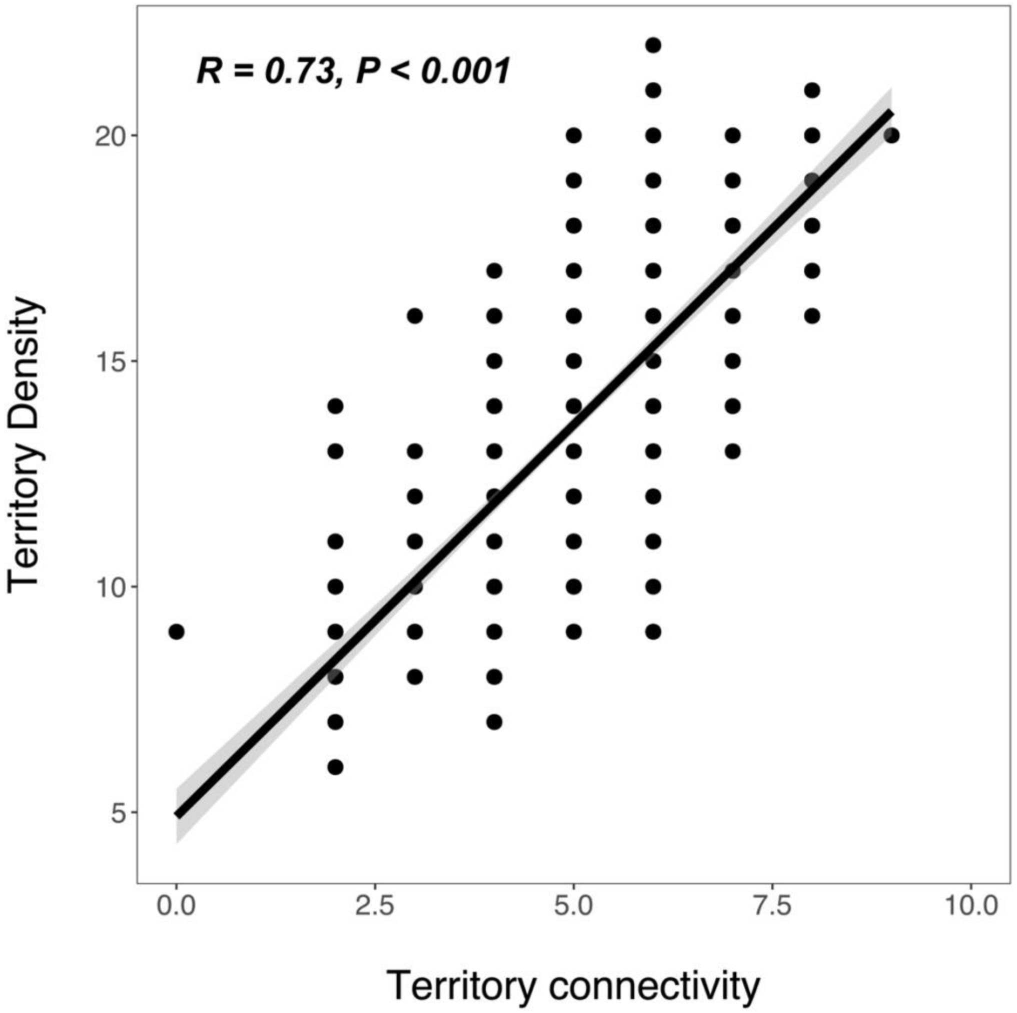

Figure S2. The relationship between geographic distance (in metres) and Seychelles warbler gut microbiome Aitchison distance (beta diversity) in each sample year. Points represent the mean (± SE) Aitchison distance per 50 m and are calculated from the raw data. Numbers at the top of each panel represent the number of pairwise comparisons contributing to each mean. Total N = 2076, 6945, 3066, 2483, 6729, and 6031 in each of 2017, 2018, 2019, 2020, 2021, and 2022, respectively. Black lines are the model predicted slopes ± 95% CI from a Multiple Regression on distance Matrices (MRM) model (see Table S2 for full results).

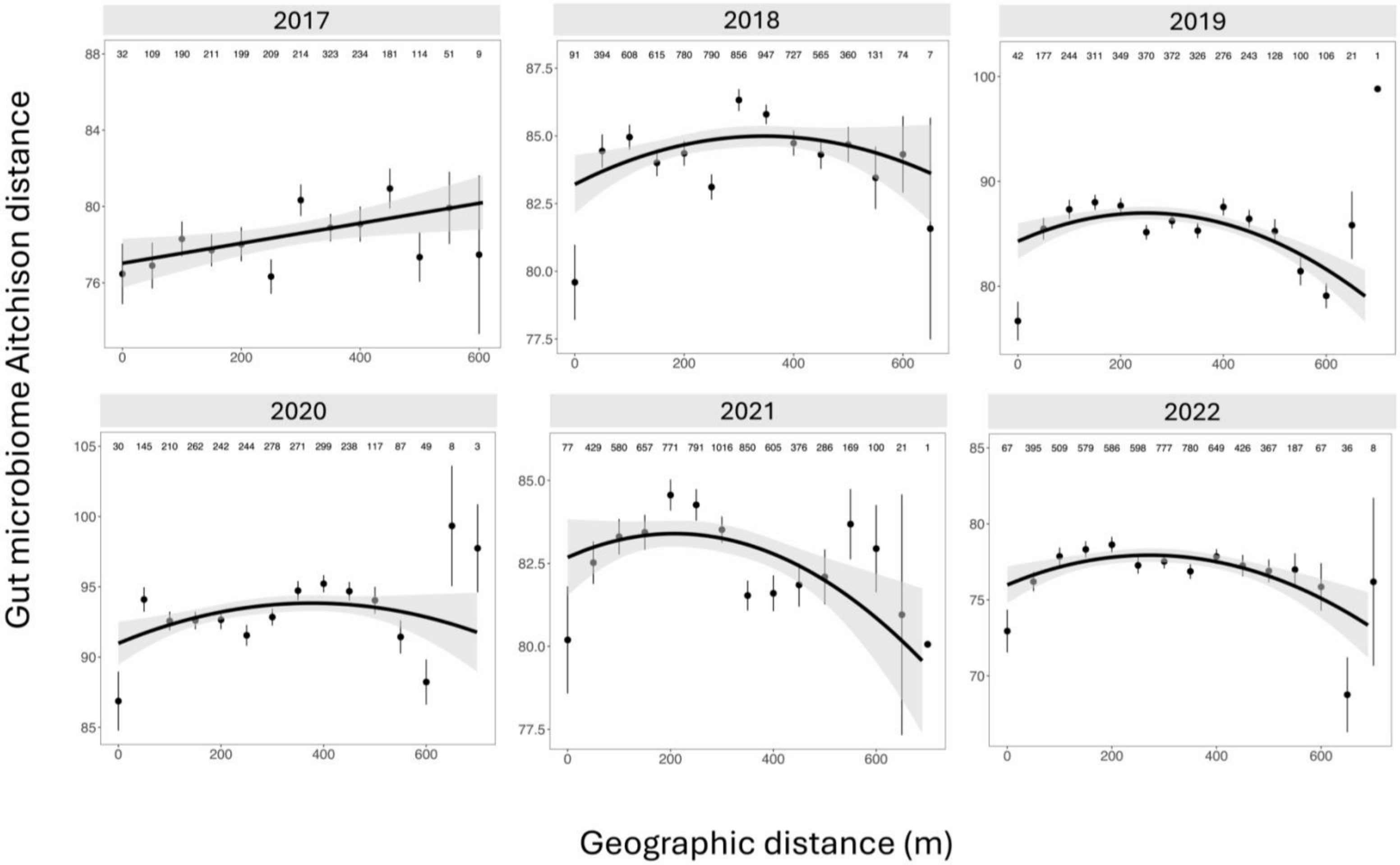

Figure S3. The relationship between geographic distance (in metres) and Seychelles warbler gut microbiome Aitchison distance (beta diversity) in each sampling season. Points represent the mean (± SE) Aitchison distance per 50 m and are calculated from the raw data. Numbers at the top of each panel represent the number of pairwise comparisons contributing to each mean.. Black lines are the model predicted slopes ± 95% CI from a Multiple Regression on distance Matrices (MRM) model (see Table S3 for full results).

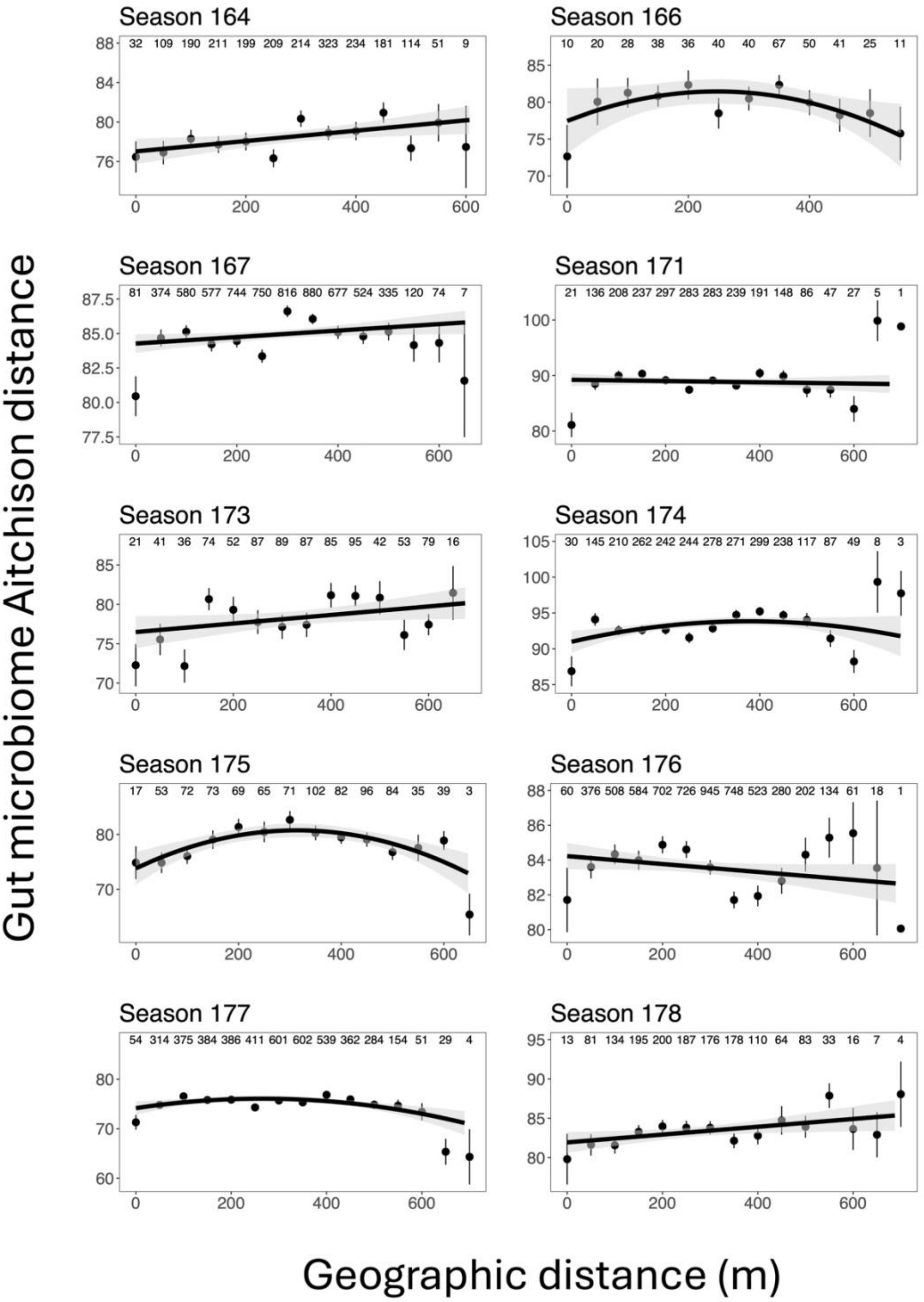

Figure S4. The relationship between geographic distance (in metres) and pairwise host genetic relatedness in Seychelles warblers. Points represent the mean (± SE) host relatedness per 50 m and are calculated from the raw data. Numbers at the top of each panel represent the number of pairwise comparisons contributing to each mean. Total N = 27,330 pairwise comparisons between 691 samples (from 390 individuals). Black lines are the model predicted slopes ± 95% CI from a Multiple Regression on distance Matrices (MRM) model (permuted *P*-value for geographic distance^2 = 0.001).

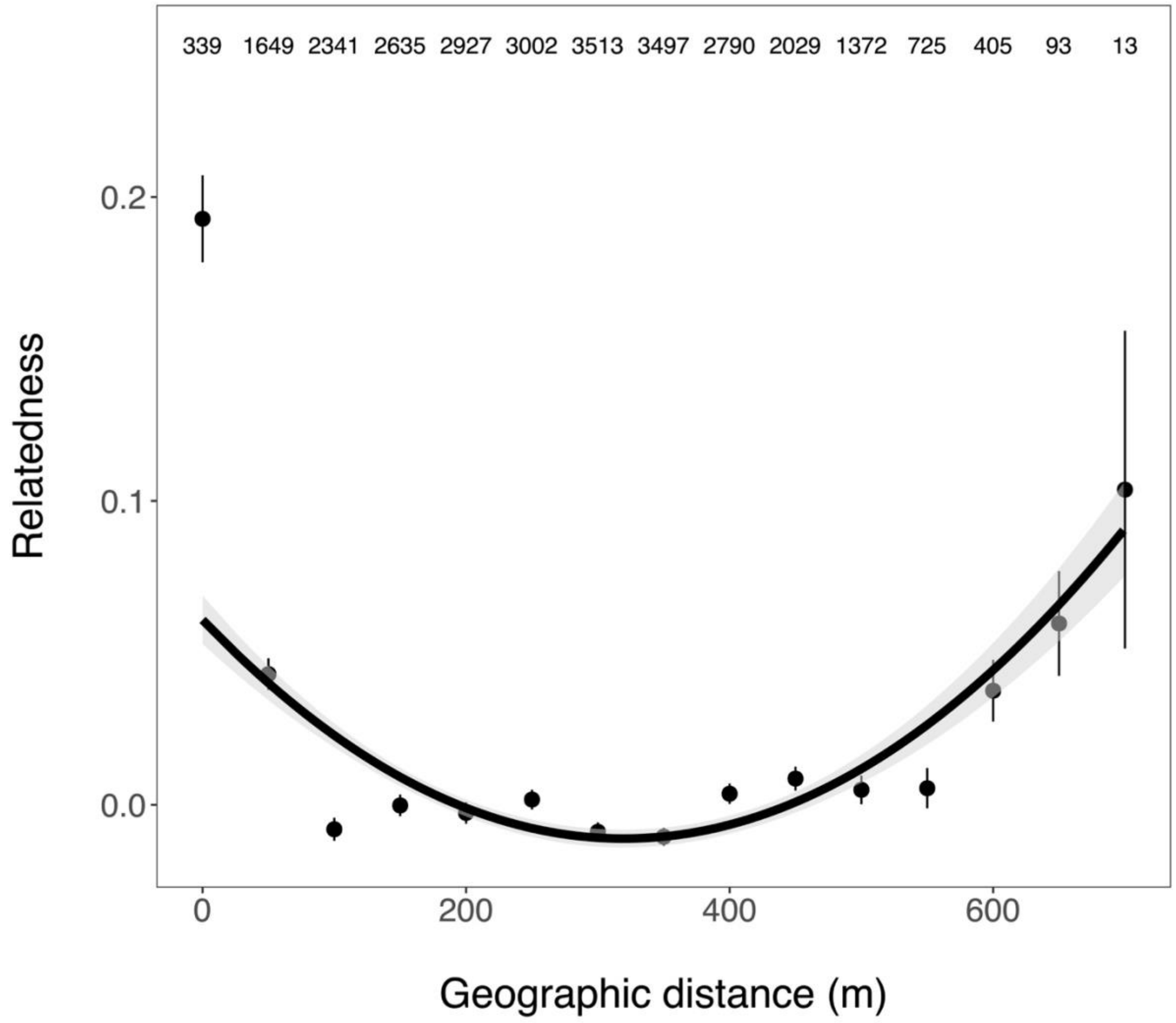

Figure S5. The relationship between geographic distance (in metres) and gut microbiome Aitchison distance (beta diversity) across A) inland and B) coastal territories of the Seychelles warblers. Points represent the mean (± SE) Aitchison distance per 50 m and are calculated from the raw data. Numbers at the top of each panel represent the number of pairwise comparisons contributing to each mean. Total N = 11,522 and 3587 pairwise comparisons between A) inland and B) coastal territories, respectively. Black lines are the model predicted slopes ± 95% CI from a Multiple Regression on distance Matrices (MRM) model (permuted *P*-value = 0.004 and 0.001, respectively – see Table S5).

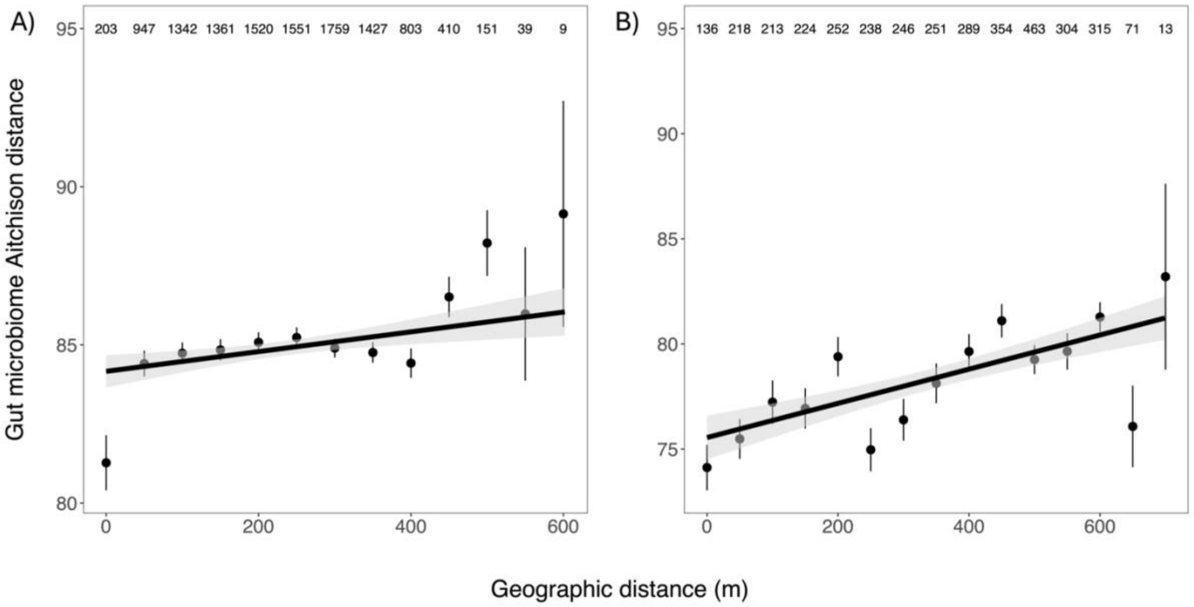

Figure S6. Differences in gut microbiome compositional variability (distance to centroid) amongst Seychelles warblers inhabiting different habitat types. Boxes encompass the interquartile (25-75%) range, the median is marked by a horizontal line and whiskers extend to 1.5x the interquartile range. Lines and numbers show the permuted *P*-value of pairwise comparisons. Significance was assessed using a Betadisper test. Significant differences in gut microbiome variation are indicated with *.

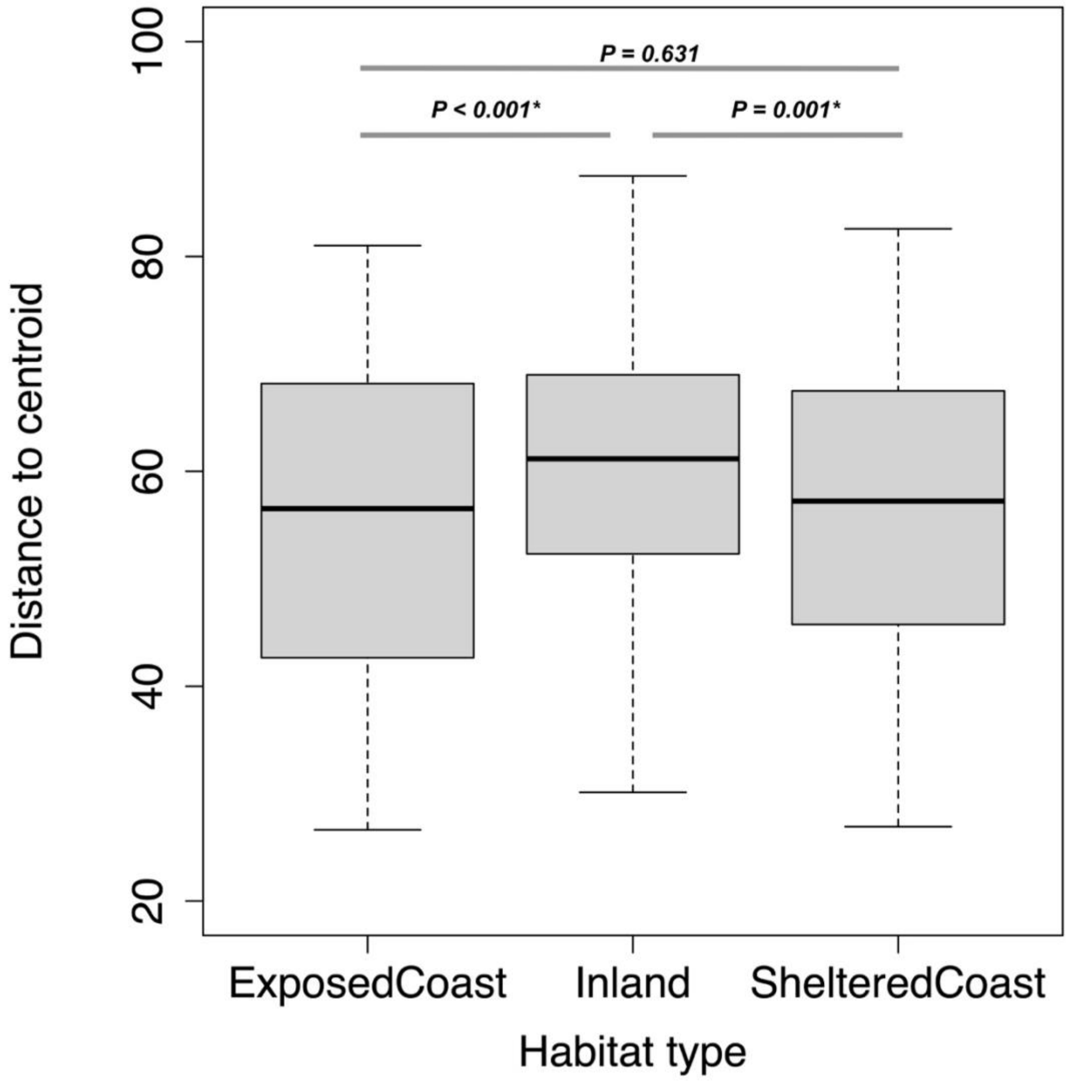

Figure S7. Variation in Seychelles warbler gut microbiome composition according to the sampling year. PCA ordination was carried out using Aitchison distances calculated on Centred Log Ratio (CLR)- transformed amplicon sequencing variant (ASV) abundances. Each point represents a unique gut microbiome sample (N = 691 samples from 390 individuals). Large diamonds represent the year centroids and ellipses are the standard deviation of each centroid. Principal components 1 and 2 explained 10.67% and 4.66% of the variation in gut microbiome composition, respectively.

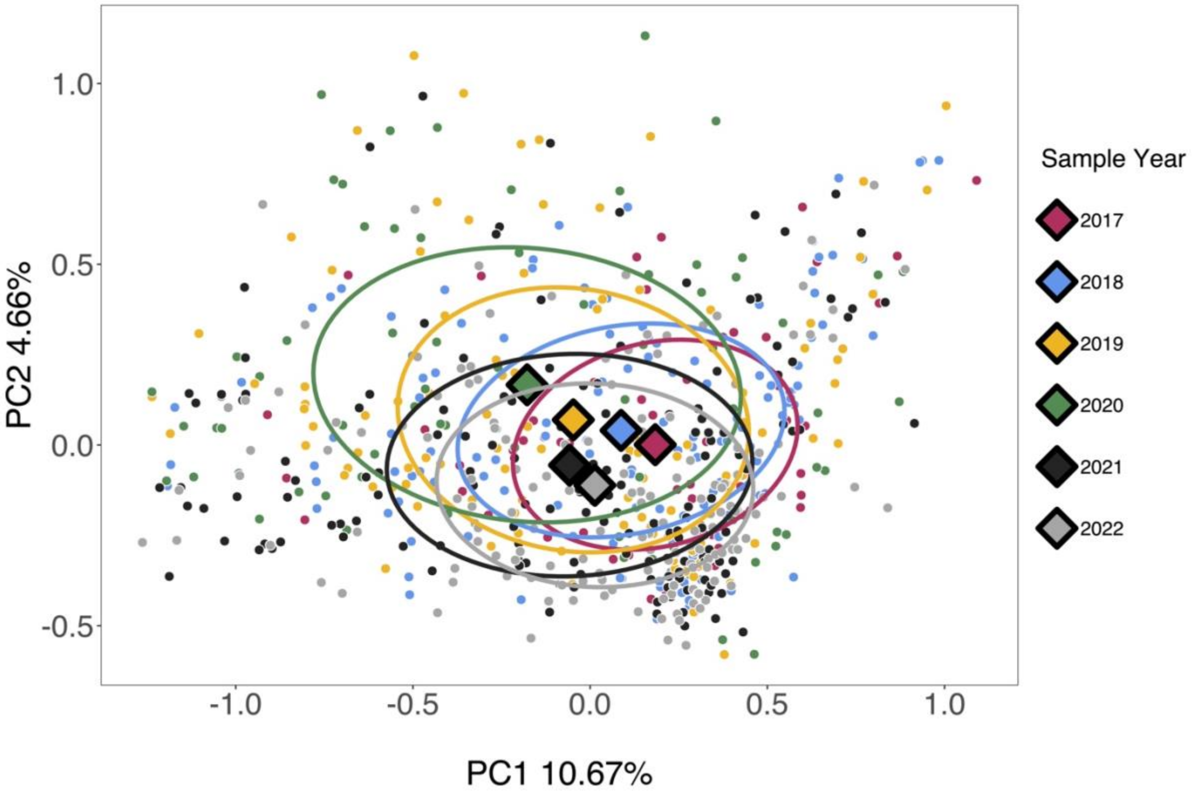

Supplement: FinalSupplementary20250802_ycaf138 [file finalsupplementary20250802_ycaf138.pdf]
